# Advance care planning readiness, barriers, and facilitators among seriously ill Black older adults and their surrogates: A mixed methods study

**DOI:** 10.1017/S1478951524001548

**Published:** 2025-01-14

**Authors:** Rebecca Howe, Shreya Kumar, Laura Slattery, Stephanie Milton, Orly Tonkikh, Everlyne G. Ogugu, Julie T. Bidwell, Janice Bell, Grace Amadi, Alicia Agnoli

**Affiliations:** 1 Department of Family Medicine, Warren Alpert Medical School of Brown University, Providence, RI, USA; 2 VA Providence Healthcare System, THRIVE Center of Innovation (COIN), Providence, RI, USA; 3 Department of Family and Community Medicine, University of California, Davis, CA, USA; 4 School of Medicine, University of California, Davis, CA, USA; 5 Department of Religious Studies, University of California, Davis, CA, USA; 6 Betty Irene Moore School of Nursing, University of California, Davis, CA, USA

**Keywords:** Advance care planning, caregivers, ethnic and racial minorities, mixed methods

## Abstract

**Objectives:**

Advance care planning (ACP) supports communication and medical decision-making and is best conceptualized as part of the care planning continuum. Black older adults have lower ACP engagement and poorer quality of care in serious illness. Surrogates are essential to effective ACP but are rarely integrated in care planning. Our objective was to describe readiness, barriers, and facilitators of ACP among seriously ill Black older adults and their surrogates.

**Methods:**

We used an explanatory sequential mixed methods study design. The setting was 2 ambulatory specialty clinics of an academic medical center and 1 community church in Northern California, USA. Participants included older adults and surrogates. Older adults were aged 60+, self-identified as Black, and had received care at 1 of the 2 clinics or were a member of the church congregation. Surrogates were aged 18+ and could potentially make medical decisions for the older adult. The validated ACP engagement survey was used to assess confidence and readiness for ACP. What “matters most” and barriers and facilitators to ACP employed questions from established ACP materials and trials. Semi-structured interviews were conducted after surveys to further explain survey results.

**Results:**

Older adults (*N* = 30) and surrogates (*N* = 12) were confident that they could engage in ACP (4.1 and 4.7 out of 5), but many were not ready for these conversations (3.1 and 3.9 out of 5). A framework with 4 themes – illness experience, social connections, interaction with health providers, burden – supports identification of barriers and facilitators to ACP engagement.

**Significance of results:**

We identified barriers and facilitators and present a framework to support ACP engagement. Future research can assess the impact of this framework on communication and decision-making.

## Introduction

As recent literature has called into question the value of advance care planning (ACP), (Morrison et al. 2021) focus has shifted to conceptualizing ACP as part of a continuum of care planning that emphasizes preparation for communication and medical decision-making. (Hickman et al. 2023) Static documents like advance directives are too limited in scope and rarely capture values and what matters most to adults with serious illness. (McMahan et al. 2021) Identifying “what matters most” is a priority of age-friendly health system transformation. (Burke et al. 2022) As a process, however, ACP has had variable uptake among different population groups.

Racially minoritized populations, including Black older adults, have significantly lower rates of ACP as well as lower quality end-of-life care. (Harrison et al. 2016; Sanders et al. 2016) While the reasons for lower ACP rates and quality of care are varied, provider bias in initiating conversations has recently been reported as a potential cause of less ACP engagement of minoritized populations. (Ashana et al. 2021) Community-based participatory research can promote equity and has been shown to increase ACP engagement among Black older adults. (Nouri et al. 2023) Surrogate engagement is also essential to ACP, and understanding the perspectives of both older adults and surrogate care partners can facilitate more effective care planning. (Fried et al. 2017).

Choosing a surrogate and having conversations about what matters most can be difficult for even the closest of patient–surrogate dyads. Just as conversations about health behavior change can be targeted to an individual’s readiness to consider and enact changes, conversations about ACP can be guided by confidence and readiness, assessed in a standardized survey. (Fried et al. 2009; Schickedanz et al. 2009) For this study, we focused on a population of Black older adults with serious illness and their surrogates, with a primary goal of describing readiness, barriers, and facilitators of ACP.

## Methods

### Design overview

**Figure 1.**
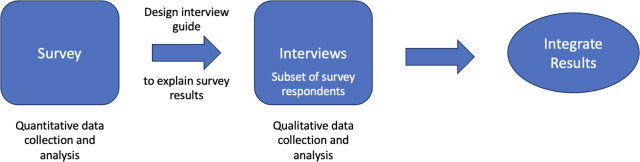
Explanatory sequential design.

Given the complexity of ACP as a process, the impact of which can only be partially captured through existing outcome measures, (McMahan et al. 2021) we selected an explanatory sequential mixed methods design to allow for further understanding of initial quantitative results (Fig. 1). (Fetters et al. 2013)

Participants first completed a survey, with the results informing the design of a semi-structured interview guide. Interviews were then completed with a subset of survey respondents. Surveys and interviews were completed from August 2022 until May 2023. Study design and procedures were reviewed and determined to be exempt by the University of California, Davis institutional review board (ID 1778912–1), and all participants provided verbal informed consent to take part in the study.

### Setting

Participants were enrolled from 2 health clinic settings and 1 community church setting in a major urban center of Northern California. The clinic settings were an advanced heart failure clinic and a geriatric clinic which serves older adults with cognitive, functional, or caregiving concerns not able to be managed in primary care. These populations align with our focus on a seriously ill population, using Kelley et al.’s definition of serious illness as “a health condition that carries a high risk of mortality AND either negatively impacts a person’s daily function or quality of life, OR excessively strains their caregivers.” (Kelley and Bollens-Lund 2018)

### Participants and recruitment

For clinic enrollment, English-speaking adults aged 60+ who self-identified as Black or African American and had a visit at the clinic in the past year received online patient portal study invitations followed by mailed card and phone invitations. Eligible older adults were asked to identify a potential surrogate (someone who could make future medical decisions for them) who was then contacted by phone. Older adults who were eligible and interested in participating but were unable to (or chose not to) identify a surrogate were still enrolled in the study.

For church enrollment, announcements about the study were made at church services and a posting was included in the congregation newsletter. A study team member provided study information in-person following church services. Interested congregation members called the study phone line to participate.

### Quantitative data

Primary outcome data were collected by electronic (REDCap [Harris et al. 2009]) or paper survey (depending on participant preference) and included instruments to measure ACP readiness and associated barriers and facilitators to readiness. (Curtis et al. 2018; IHI TCP 2021; Sudore et al. 2017) Additional quantitative data were collected on sociodemographics, health status and comorbidities, (EuroQol Research Foundation 2018; Quan et al. 2011) spirituality, (Underwood and Teresi 2002) and loneliness. (Russell 1996)

### Qualitative data

Participants who completed the survey were asked if they would be willing to be contacted in the future for an interview to further discuss their responses. The semi-structured interview guide (see **Appendix**) was developed based on initial survey results as well as key domains from a related study. (Lopez et al. 2021)

### Data analysis

Quantitative survey data were analyzed descriptively using STATA 15 (StataCorp, College Station, Texas). For dyads, congruence between older adult and surrogate readiness and confidence were assessed visually using dyadic spaghetti plots. Interviews were recorded and transcribed with 4 study researchers (RH, SM, OT, EO) initially reviewing the same 2 transcripts and using both deductive coding (from interview guide domains [Lopez et al. 2021]) and inductive coding to develop a preliminary codebook. The codebook was refined after the same study researchers independently coded 4 additional transcripts followed by meetings to review and come to consensus on the codebook. This codebook was then applied to the remaining transcripts by the same 4 study researchers. Memos were documented to capture reflective, analytical, and methodological insights and changes to support the research audit trail. Thematic analysis was used to analyze the transcripts. Quantitative and qualitative data were integrated through the study design, dyadic analyses, and narrative integration of themes. We followed current mixed methods manuscript preparation and reporting guidelines. (Lee et al. 2022)

## Results

### Quantitative data

**Figure 2.**
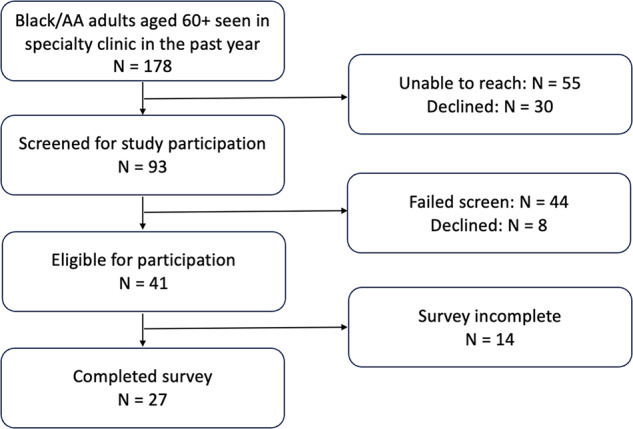
Health center enrollment flow diagram.

Survey participants were 30 older adults and 12 surrogates and included 11 dyads. Figure 2 displays the enrollment process for the older adults from the health clinics. Table 1 provides additional participant characteristics.

**Table 1.** Participant characteristics

Participant characteristics	Number of older adults *N* = 30	Number of surrogates *N* = 12
Age		
*Median (Range)*	72 (60–91)	62 (38–92)
Gender		
*Female*	18 (60%)	11 (92%)
Ethnicity		
*Hispanic or Latino*	0	2 (17%)
Race		
*African American or Black*	26 (87%)	9 (75%)
*Other*^*a*^	4 (13%)	5 (42%)
Comorbidities		
*At least 1 severe comorbidity*^*b*^	21 (70%)	1 (8%)
Subjective health		
*EQ-VAS median (range)*^*c*^	62 (14–100)	80 (50–94)
Surrogate relationship to older adult		
*Child*		7 (58%)
*Spouse/Partner*		5 (42%)
Spirituality		
*Self-identify as at least somewhat spiritual*	28 (93%)	11 (92%)
Education level		
*High school or less*	6 (20%)	1 (8%)
*Some college*	9 (30%)	7 (58%)
*2-year college degree*	4 (13%)	0
*4-year college degree*	4 (13%)	4 (33%)
*Postgraduate*	7 (23%)	0
Marital status		
*Married*	11 (37%)	7 (58%)
*Living with a partner*	1 (3%)	0
*Separated*	8 (27%)	2 (17%)
*Divorced*	5 (17%)	1 (8%)
*Single (never married)*	4 (13%)	2 (17%)
Place of residence		
*Private residence*	28 (93%)	12 (100%)
*Assisted living*	1 (3%)	0
Cohabitation		
*Lives alone*	9 (30%)	0
*Lives with 1 other person*	12 (40%)	5 (42%)
*Lives with 2 or more other people*	8 (27%)	7 (58%)
Lonely^d^	8 (27%)	3 (25%)
Enrollment site		
*Healthy aging clinic*	11 (37%)	
*Heart failure clinic*	16 (53%)	
*Church community*	3 (10%)	

a Other race refers to Creole and mixed for older adults, and Asian, Caucasian, Creole, Mexican, and Puerto Rican for surrogates.

b A severe comorbidity was defined as self-reporting a history of emphysema, chronic bronchitis, or other chronic lung disease, cancer, heart attack, heart failure, or stroke.

c EQ-VAS is a self-assessment of health where participants rate their overall health on a scale of 0–100, with 0 being the worst imaginable state of health and 100 being the best imaginable state of health.

d Based on the UCLA Loneliness Scale, version 3. Scores ranged from 4–12 and participants were considered to be lonely if they had a score greater than 7.

Older adults and surrogates were confident they could engage in ACP (4.1 and 4.7 out of 5), but many were not ready for these conversations (3.1 and 3.9 out of 5). On surrogates’ surveys, “confidence” refers to their confidence in serving as a surrogate, talking with the older adult, and talking with the older adult’s doctor. When asked to list what matters most to them, including activities that bring meaning or joy, the most common response among older adults was “not being a burden on your family” (90%), followed by “your family or friends” (87%). When surrogates responded what they thought was most important to the older adult, the most common response was “their family or friends” (100%), followed by “hobbies, such as gardening, reading, cooking” (75%). Only 33% of surrogates thought that the older adult worried about being a burden.

**Figure 3.**
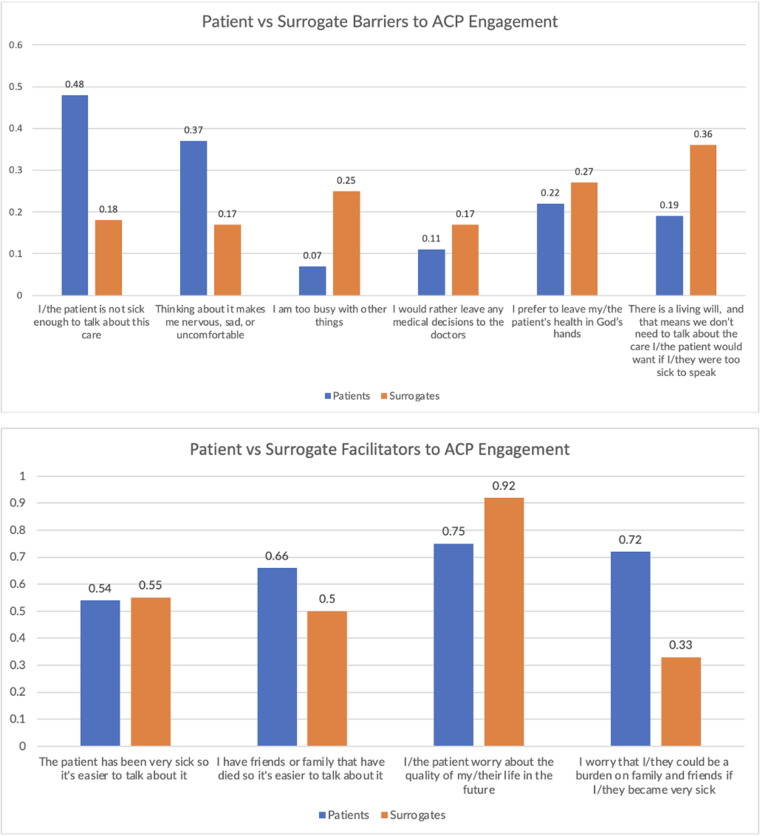
Barriers and facilitators of ACP engagement.

Figure 3 presents the barriers and facilitators of ACP for both older adults and surrogates. Participants were asked to agree or disagree with several statements about what makes talking harder and easier. Patients (older adults) were most likely to list not being sick as a barrier and worry about quality of life/being a burden as a facilitator, whereas surrogates were most likely to list having a living will as a barrier and worrying about the older adult’s quality of life as a facilitator.

Proportion of older adults and surrogates who agree with the statement provided.

### Dyadic plots

**Figure 4.**
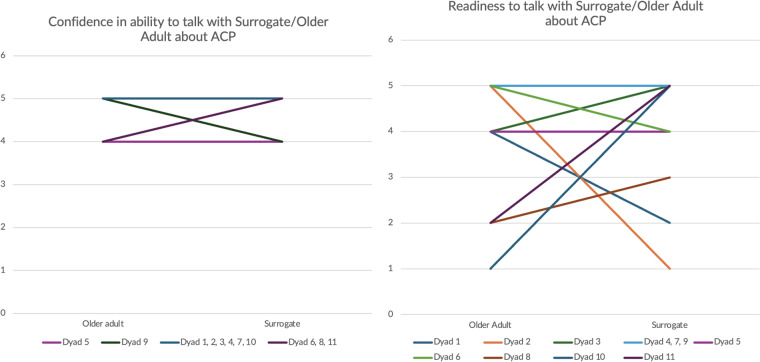
Confidence and readiness for ACP.

Figure 4 presents plots of dyadic congruence in confidence and readiness to discuss ACP. In these plots, each line represents 1 (or more) dyads, with each older adult’s score (left side of the plot) connected to their surrogate’s score (right side of the plot). We observed generally congruent responses in dyads related to confidence to discuss ACP (Fig. 4a), but more variability and greater incongruence in dyads’ readiness to discuss ACP with each other (Fig. 4b).

Confidence (Fig. 3a) and Readiness (Fig. 3b) to talk about ACP, where y-axis is the mean score on a Likert 5-point scale. Horizontal lines represent congruence and steeper sloped lines represent greater incongruence between the older adult and surrogate.

Interviews were subsequently conducted with 16 participants (12 older adults and 4 surrogates and included 3 dyads).

### Qualitative data

**Figure 5.**
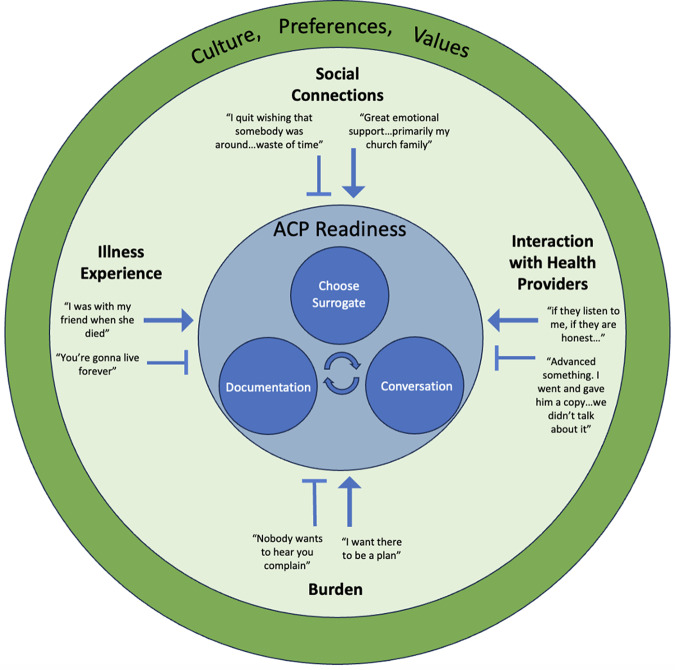
Concept map of the barrier and facilitator themes of ACP engagement.

Figure 5 presents a concept map of the qualitative analysis, with ACP readiness surrounded by the 4 predominant barrier and facilitator themes, all within the broader context of culture, preferences, and values. Explanatory quotes for each theme and the context are included in Table 2.

**Table 2.** Theme and context quotes

Theme	Exemplary quotation
ACP barriers and facilitators	
– Illness experience	“I didn’t think I should bring it up … because my health is good … I don’t even know how to start the conversation.” “All of a sudden, I’m in the hospital … I was thinking, Oh my God, I didn’t do this planning. I don’t have a will. I don’t have a trust. So … I hope it doesn’t take that for other people.”
– Social connections	“They say, ‘Mom, that’s not right, here we are, we could do it.’ And I tell them all the time, there will be a time when I cannot pay someone to clean the house. That’s when I want you to say, okay, but if I use you up now, when comes that time, you’ll be too tired.” “[If] you don’t talk with people, they don’t know what’s best for you. It’s like guessing. So you wanna take the guesswork out, and [add] a little bit of knowledge.”
– Interactions with health providers	“Yeah, advanced. Advanced something. I went and gave him a copy, and then he said they would put it in my chart … That was it. We didn’t talk about [it].” “If they listen to me, if they are honest, if they are – if they give me information, and if they are very keen in knowing that I’m looking at them from the standpoint of a Black man … I’ve had experiences with doctors that made me feel like they found me underneath a rock. I will not work with doctors that do that.”
– Burden	“… because I really don’t want to be a burden to my friends. You will certainly lose friendships that fast … like I said, you don’t want to wear anyone out.” “Decision making is hard. And so all of the things I had to do, [or the booklet that I was talking about that I gave my cousin], had to be thought over, prayed over, concentrated, ripped up, started over.” “Honestly, I wasn’t raised like that. I love my mom dearly with all my heart. There is nothing on God’s earth that she could do, or experience that would be a burden for me ever.”
Context of culture, value, preferences	“I’m African American. And in our culture, we’re not raised to think about life insurance. We’re just not and so a lot of times when people pass, you know, people have to pass around, before the GoFundMe, you would pass around the hat … Again, hence, a burden on the living because the old man used to tell me all the time, ‘funerals are not for the dead, funerals are for the living.’ And so it was always, you know, the responsibility of the loved ones to take care of the departed … I didn’t want that to happen for me … When I go, I want there to be a plan.” “No, I don’t want someone to have to take care of me 24 hours a day. That’s not quality of life. That’s not me. I don’t want to die, but if I’m just living to live, that’s not life to me. And I don’t want to do that. And I guess mainly, it’s because my religious belief, too, that I believe that death is merely a sleep. So I would rather just sleep.”

#### ACP readiness

Participant interviews focused on 3 aspects of ACP readiness: choice of surrogate, conversations, and documentation. Conversations included those with family or friends as well as health providers. Documentation typically referred to advance directives. Participants did not describe a particular order to the ACP components, with some starting with choice of surrogate, others with conversations. The arrows between the 3 components of ACP readiness in Fig. 5 depict this nonlinear, iterative process. When describing choice of surrogate, proximity and close relationship were important factors. Often a family member or friend was selected as surrogate, but several participants mentioned choosing a religious leader.

#### ACP barriers and facilitators

The 4 predominant barrier and facilitator themes were 1. Illness experience, 2. Social connections, 3. Interaction with health providers, and 4. Burden. Figure 5 displays how each theme could represent either a barrier or a facilitator of ACP readiness.

##### Illness experience

Illness experience includes both health status and perception of health. For 1 participant, self-perception of good health was a barrier to ACP. For another participant, her personal past experience with acute serious illness was a facilitator for ACP.

##### Social connections

Participants referred to social connections when discussing current or future needs for care. One older adult participant explained her decision not to have her daughters support her with care currently. Social connection also related to discussions with surrogates around care preferences.

##### Interaction with health providers

Participants often referenced interactions with health providers as abbreviated and focusing on documentation. One participant shared how previous experiences of racial discrimination impacted his interaction with health providers.

##### Burden

Burden was discussed in several contexts, including the physical burden of care related to instrumental activities and activities of daily living. Participants discussed the financial burden of care, with still others reporting a cognitive burden related to decision-making. Several participants described the ACP conversations as being a burden. Surrogates were less likely to state that caring for a family member would be a burden.

#### Context of culture, values, and preferences

ACP engagement was often discussed in relation to sociocultural contexts. Among our sample, racial identity, religious beliefs, and familial values impacted care planning and decision-making.

## Discussion

Black older adults with serious illness and their surrogates were confident that they could engage in ACP, but many were not yet ready. We present a framework for ACP engagement with 4 barrier and facilitator themes – illness experience, social connections, interaction with health providers, and “burden” – which when considered in the context of culture, values, and preferences, offers opportunity for further ACP engagement. We discuss each component of the framework and implications for ACP facilitation below.

### ACP readiness

Our finding that participants were confident that they could engage in ACP but felt less ready is similar to findings by Li et al., (Li et al. 2024) who also found that surrogates were even more confident than older adults that they could talk about ACP. Li et al. discuss that surrogate overconfidence can be a barrier to ACP and that studies have shown no correlation between surrogate confidence and knowledge of patients’ wishes. (Green et al. 2018) In both our survey and interviews, participants had diverse perspectives on what constitutes ACP, reflective of the changing definitions and context around ACP in recent years. Surrogates were more likely than older adults to agree with the statement, “there is a living will, and that means I don’t need to talk about the care they [older adult] would want.” Auriemma et al. found that focusing on documentation such as advance directives and living wills can undermine ACP, as individuals demonstrate a “set it and forget it” mentality that lessens their willingness to continuously engage in conversation and decision-making as health changes. (Auriemma et al. 2022) More work is needed to help patients and surrogates understand ACP as part of the care planning umbrella, (Hickman et al. 2023) a process that assists with communication and decision-making rather than a static document. Some have proposed new terminology to capture this evolving understanding of ACP, with “AdaptCP” as a term that highlights that ACP and medical decision-making should adapt as information and conditions change. (Moody et al. 2024) Exploring the 4 barrier and facilitator themes below may help older adults and surrogates progress along the spectrum of readiness to full ACP engagement.

### ACP barriers and facilitators

Burden, illness experience, social connections, and interaction with health providers were the 4 themes that were frequently raised by both older adult and surrogate participants in our survey and interviews. As highlighted in Fig. 5, each theme could serve as a barrier or facilitator of ACP readiness.

#### Burden

We were surprised to find that “not being a burden” was what matters most to older adults, despite previous research in African American populations finding that the term “burden” was misaligned with caregiving experiences. (Brewster et al. 2020) Concern that the older adult would be a burden was raised less often by surrogates, and our interviews did support a cultural value of familism and filial piety. (Brewster et al. 2020) Older adults spoke to many aspects of burden: physical strain of activities of daily living care, financial stress, cognitive burden of decision-making, emotional burden of conversations focused on end of life. Many of these care tasks considered burdensome can be alleviated with proactive connections to resources, and previous research has shown that marginalized populations have lower levels of preparedness for care planning. (Li et al. 2024) Both older adults and surrogates were similarly concerned about the older adult’s quality of life in the future. Focusing on language of stress/strain and quality of life – and on supporting success with caregiving activities rather than the older adult as an individual being a burden – may facilitate ACP engagement.

#### Illness experience

Past personal or caregiving experience with serious illness was frequently cited as a facilitator of ACP engagement. However, older adults were more likely than surrogates to report they were “not sick enough” as a reason for not engaging in ACP. Many of the older adults were enrolled from a heart failure clinic, and heart failure illness perception can be particularly variable due to the waxing and waning trajectory of heart failure symptoms. (Allen et al. 2012) Understanding and integrating prognostic information cognitively and emotionally is a challenge of ACP engagement, (Jackson and Emanuel 2023) and discussing care with older adults and surrogates together using established conversation frameworks (e.g. serious illness conversations (Ariadne Labs: A Joint Center for Health Systems Innovation (https://www.ariadnelabs.org) between Brigham and Women’s Hospital and the Harvard T.H. Chan School of Public Health in collaboration with Dana-Farber Cancer Institute 2023) or VitalTalk (“Home – VitalTalk” n.d.) may facilitate accurate, congruent prognostic understanding.

#### Social connections

Connections with family, friends, neighbors, and church community were often raised during interviews in relation to presence or absence of support as well as readiness for ACP. Social isolation and loneliness are associated with poor health and early mortality, (Wang et al. 2023) but when considering social connections, it is important to assess not only whether an individual has connections but also the quality and function of those social connections. ([OSG] 2023) An older adult may have regular conversations with a friend or acquaintance, but if they never discuss values or preferences for care, it may be difficult for that individual to help make medical decisions.

#### Interaction with health providers

Participants frequently commented that health providers focused on documentation such as advance directives rather than conversations about values and preferences. While there are many programs to train providers on facilitation of conversations that focus on what matters most rather than hypothetical future procedural decision-making, (Ariadne Labs: A Joint Center for Health Systems Innovation (https://www.ariadnelabs.org) between Brigham and Women’s Hospital and the Harvard T.H. Chan School of Public Health in collaboration with Dana-Farber Cancer Institute 2023; (California 2022; Curtis et al. 2018); “Home – VitalTalk” n.d.; “Respecting Choices | Person-Centered Care” n.d.) these programs may not be prioritized when health systems incentivize the quick completion of documents instead. (CMS 2015) Care quality metrics that better align with ACP as a process – such as a recent measure about feeling “heard and understood” by the care team (Edelen et al. 2022) – may incentivize the provider training needed for ACP engaged conversations and not just documentation.

### Context of culture, preferences, and values

More than 90% of both older adult and surrogate participants described themselves as spiritual or religious, and faith communities were often referenced as sources of support, including choosing a pastor as a surrogate. Community based initiatives – such as Alter Dementia (“Home – Alter” n.d.) and the Alameda County Care Alliance (“Alameda County Care Alliance” n.d.) – leverage these strong faith communities for ACP engagement among Black older adults and surrogates. Discussions of quality of life and values require contextual awareness of racial injustices in medicine, and trusting relationships are best built with conversations over time, whether in healthcare or community settings. When discussing values and preferences, many participants desired continued independence and to reside at home as long as possible. For caregivers and health providers to feel comfortable discussing values and preferences, there need to be actionable supports to help older adults achieve these goals. The pandemic public health emergency expanded access to home and community based services in many states, (Burns et al. 2023b) but there are still long waitlists and these services are typically only available to Medicaid recipients. (Burns et al. 2023a)

Our study has several limitations. We struggled to enroll from the church community, despite early engagement with the church pastor and study design adaptations to maximize participant privacy. According to the pastor, the cognitive screening step may have been a barrier to survey completion, and it is worth considering whether this is necessary for future research in community settings. Another limitation was that few dyads completed interviews. Interviews were conducted with older adults and surrogates independently to allow for free responses without bias from being in the presence of the care partner. Future research would also benefit from considering care networks and not dyads alone, as multigenerational kinship is a strength of African American families. (Brewster et al. 2020) Finally, we were unable to assess responses longitudinally to determine whether participants’ level of readiness corresponded to actual ACP engagement later on.

The strengths of our study included the mixed methods design, which allowed for nuanced discussion of the complexity surrounding barriers and facilitators of ACP readiness. A recent study on ACP and goal-concordant care by Lenko et al. calls for more focus on the barriers and facilitators of ACP among Black older adults. (Lenko et al. 2024) Enrolling both older adults and surrogates enabled us to assess congruence and incongruence across readiness, barriers, and facilitators. One area of incongruence, burden, was explored in detail and found to be multidimensional, including physical, financial, cognitive, and emotional aspects. As this is a topic that “matters most” to many older adults, the thick description of burden in this study contrasts with the more limited information that might be gathered with a short survey instrument.

Achieving age-friendly health system transformation requires a focus on equity and investment in determining and supporting what “matters most” to Black older adults and their surrogates. Our study on ACP readiness, barriers, and facilitators highlights social connection, illness experience, interactions with health providers, and the topic of “burden” – considered in a context of culture, values, and preferences – as focus areas for the future. Training for health providers should clarify ACP as a process of conversations and not just advance directive documents, though opportunities for ACP outside of typical health systems, such as in faith communities, should also be explored. This structured framework, along with advocacy for additional resources to support aging at home, allow for the planning necessary for healthy aging and caregiving for those with serious illness.

## Supporting information

Howe et al. supplementary material Howe et al. supplementary material

## Supplementary material

The supplementary material for this article can be found at https://doi.org/10.1017/S1478951524001548.
