# Supplementary material for: Advance care planning readiness, barriers, and facilitators among seriously ill Black older adults and their surrogates: A mixed methods study
Source: Palliat Support Care. 2025 Jan 14;23:e15. doi: 10.1017/S1478951524001548 (PMC13168772; doi:10.1017/S1478951524001548)
Supplement: Howe et al. supplementary material [file S1478951524001548sup001.docx]

**Advance Care Planning Semi-Structured Interview Questions**

Thank you for taking the time to talk with me today. This interview should take about 45 minutes. We’ll talk about how you make some healthcare decisions, what’s important to you, where you find support, and how you prefer to talk about these topics with others. These questions are part of a research study, and your participation is voluntary. You do not have to answer any questions you don’t want to and you can also stop at any point. We will be recording the interview, but there will be no identifying information, such as name or birthdate, linking you to the audio recording. You’ll receive a $40 gift card after the interview to thank you for your time. If you have any questions about this research, you can contact the study investigator at xxx-xxx-xxxx. Would you like to participate?

*Decision-making process:*

1. First, I’d like you to think about an important healthcare decision you’ve made recently, or maybe something you’re still considering but haven’t yet decided. This could be about whether or not to seek help for an illness or symptom, a decision about whether to have a type of treatment or test, a decision about where to receive healthcare (at home or in the hospital or in a rehab facility), or a decision about who will provide needed care. Take a moment to think about this. Do you have something in mind? Okay great, tell me about that healthcare decision.
2. How did you go about making that decision?
   1. Lots of things can play a role in how we make healthcare decisions, such as previous experiences, social connections, religion and spirituality. Were any of these important in your decision?
3. Another important healthcare decision is determining who would be a surrogate, or the person who would make healthcare decisions for you if you weren’t able to make your own decisions. What are some things you thought about when deciding who would be your healthcare surrogate?

I’d like to hear more about any conversations you’ve had with your surrogate or with your medical team about future health decisions.

*Communication with surrogate and medical team:*

1. If you’ve had conversations with your surrogate, how did those talks go?
   1. In the survey, you checked that you feel confident you could talk with your surrogate about the kind of medical care you would want if you were very sick or near the end of life but are not yet ready to talk with them. Can you tell me more about that? What would help you feel ready to have these conversations?
2. In what setting would you feel most comfortable talking with your surrogate about these topics? I.e. at home, at church, at a doctor’s appointment…
3. Who should be included in these conversations? I.e. your surrogate, other family or friends, your pastor, your doctor…
4. What would help you feel ready to have these conversations with your doctor or medical team?

*Exploring conceptions of “being a burden”:*

In the survey, many people said that it was very important for them not to be a burden on family.

1. What would “being a burden” look like to you?
2. Was there ever a time where you felt like you were a burden on your family? How did you handle this?
3. How, if at all, does the fear of being a burden affect the way you talk or interact with your family or people who are import to you?

----------------------------------------------- Time check

I’m also curious about your support system.

*Family and community support:*

1. Tell me more about the support you get from family, friends, or your community.
2. Do you have enough support? How does support or lack of support influence how you make decisions?

Now, I’d like to hear more about your preferences and values. In the survey, you mentioned that some things such as “spending time with family and friends” or “not being a burden on family” are important.

*Preferences and values:*

1. Can you tell me more about why you checked those things? What else is most important to you at this time?
2. What are your most important goals if your health situation worsens?
3. What are some of your worries or fears when you think about your health in the future?
4. What sustains you as you go through health issues? Where do you find hope?

I’ve come to the end of the questions I have for you. Were there any things that we didn’t talk about that you wish we had? Thank you.
